# GRHL2 contributes to the maintenance of intestinal epithelial barrier integrity during LPS-induced injury

**DOI:** 10.3389/fimmu.2026.1881734

**Published:** 2026-07-15

**Authors:** Youquan Wang, Yuting Li, Lingling Bao, Junying Lu, Xinyu Li, Yao Fu, Dong Zhang, Hongxiang Li

**Affiliations:** Department of Critical Care Medicine, The First Hospital of Jilin University, Changchun, China

**Keywords:** GRHL2, intestinal epithelial barrier, intestinal permeability, junction-associated, LPS, sepsis

## Abstract

**Introduction:**

Sepsis-induced intestinal epithelial barrier dysfunction contributes to gastrointestinal injury during critical illness, yet the molecular mechanisms underlying barrier disruption remain incompletely understood. This study investigated the potential involvement of Grainyhead-like 2 (GRHL2) in lipopolysaccharide (LPS)-induced intestinal epithelial barrier injury.

**Methods:**

LPS-induced intestinal barrier injury was evaluated using Caco-2 monolayers and a mouse endotoxemia model. Barrier integrity was assessed by transepithelial electrical resistance (TEER), immunofluorescence, histological analysis, serum cytokine measurement, and FITC-dextran permeability assays. Transcriptomic analysis was performed to identify biological processes associated with LPS-induced intestinal injury. GRHL2 expression was evaluated by qRT-PCR, immunohistochemistry, and Western blotting. Gain- and loss-of-function experiments were performed using lentiviral-mediated GRHL2 overexpression and knockdown *in vivo*.

**Results:**

LPS exposure impaired epithelial barrier integrity, as evidenced by reduced TEER values, disrupted Occludin distribution, increased serum TNF-α and IL-6 concentrations, histological injury, and enhanced intestinal permeability. Transcriptomic analysis revealed marked transcriptional alterations following LPS exposure, with enrichment of biological processes related to inflammatory responses, cell–cell adhesion, epithelial development, and extracellular matrix organization. GRHL2 expression was significantly reduced in LPS-treated intestinal tissues together with decreased expression of several epithelial junction-associated molecules, including E-cadherin, Claudin-3, Claudin-4, and Occludin. Functional modulation demonstrated that GRHL2 knockdown aggravated intestinal injury and permeability, whereas GRHL2 overexpression attenuated barrier dysfunction and was accompanied by increased expression of multiple epithelial junction-associated molecules.

**Discussion:**

These findings demonstrate that GRHL2 contributes to the maintenance of intestinal epithelial barrier integrity during LPS-induced injury. The observed association between GRHL2 downregulation and impaired expression of epithelial junction-associated molecules suggests that GRHL2 may participate in the epithelial response to inflammatory injury. Further studies are required to clarify the molecular mechanisms underlying GRHL2 regulation and its role in intestinal barrier homeostasis.

## Introduction

The intestinal epithelium is a highly dynamic barrier that separates luminal microbial products from the internal milieu while allowing nutrient and fluid absorption (1, 2). In critical illness, and particularly during sepsis, this barrier is highly vulnerable to systemic inflammation, hypoperfusion, oxidative stress, and endotoxin exposure. Disruption of epithelial tight junctions increases intestinal permeability and may facilitate the translocation of microbial products, thereby amplifying systemic inflammatory responses and contributing to multiple-organ dysfunction. Despite the clinical relevance of gut barrier failure in sepsis (3), the molecular mechanisms linking inflammatory stimulation to tight junction disruption remain incompletely defined.

Lipopolysaccharide (LPS), a major component of the outer membrane of Gram-negative bacteria, is widely used to model sepsis-associated inflammatory injury. Experimental exposure to LPS can induce epithelial injury, impair tight junction organization, and increase paracellular permeability (4). However, the transcriptional regulators that connect LPS-induced inflammatory signaling with the loss of epithelial junction integrity remain insufficiently understood.

Grainyhead-like transcription factor 2 (GRHL2) is a member of the grainyhead-like family of transcription factors and has been implicated in epithelial development, differentiation, and barrier maintenance (5). GRHL2 supports epithelial identity and cell junction integrity in several tissues and has been reported to regulate junction-associated molecules, including E-cadherin, claudins, occludin, and ZO-1 (5–8). These proteins are central components of epithelial tight junctions and adherens junctions, and their disruption can increase epithelial permeability and promote barrier dysfunction. Notably, previous work has shown that Porphyromonas gingivalis can compromise oral epithelial barrier integrity by targeting GRHL2, suggesting that GRHL2 may represent a shared regulatory node in epithelial barrier injury across different mucosal tissues (7, 9). Whether a similar GRHL2-related mechanism contributes to LPS-induced intestinal barrier disruption remains unclear.

In this study, we used complementary *in vitro* and *in vivo* models to examine the role of GRHL2 in LPS-induced intestinal barrier injury. Caco-2 epithelial monolayers were used to assess barrier function and tight junction organization, while an LPS-induced mouse model was applied to evaluate intestinal injury, permeability, transcriptomic remodeling, and GRHL2-dependent regulation of junction-associated proteins. We hypothesized that LPS exposure would be associated with GRHL2 downregulation and that altered GRHL2 expression would influence intestinal epithelial barrier integrity during inflammatory injury.

## Materials and methods

### Ethics statement

All animal experiments were approved by the Animal Ethics Committee of Jilin University (Approval No. 20210594). All procedures were performed in accordance with institutional guidelines and were conducted under appropriate anesthesia and analgesia whenever applicable to minimize animal suffering.

### Cell culture

Caco-2 cells were cultured in DMEM/F12 medium supplemented with 10% fetal bovine serum (FBS) and 1% penicillin–streptomycin at 37 °C in a humidified atmosphere containing 5% CO^2^. The culture medium was replaced every 2 days. Prior to experiments, cells were serum-starved for 24 h. For barrier injury experiments, cells were stimulated with lipopolysaccharide (LPS, 10 μg/mL) for up to 48 h.

### TEER and permeability assays

To evaluate epithelial barrier integrity, transepithelial electrical resistance (TEER) was measured using differentiated Caco-2 monolayers cultured on Transwell inserts (Corning Costar, 24 mm diameter, 0.4 μm pore size). Cells were seeded at a density of 6 × 10^5^ cells/well. Cells were cultured for 7–9 days. TEER values were monitored during culture, and experiments were performed after TEER measurements reached a stable plateau, indicating the formation of a functionally mature epithelial monolayer under our culture conditions. LPS (10 μg/mL) was added to the apical compartment of the Transwell system, and TEER values were measured at 0, 12, 24, 36, and 48 h using an epithelial voltohmmeter (Millipore ESR-2, Merck Millipore, USA) before and after treatment. Blank inserts without cells were used as controls.

### Animal experiments and LPS-induced sepsis model

Male C57BL/6 mice (6–8 weeks old, 18–22 g) were maintained under SPF conditions with free access to food and water. Mice were randomly assigned to control or LPS groups. Lipopolysaccharide (LPS, Escherichia coli O111:B4) was dissolved in sterile PBS and administered intraperitoneally at 10 mg/kg. Control mice received an equal volume of PBS. Based on preliminary experiments, intestinal injury reached a near-maximal level approximately 24 h after LPS administration. Therefore, blood and tissue samples were collected 24 h after LPS injection. No mortality was observed during the 24-h observation period.

### Measurement of serum inflammatory cytokines

To confirm successful induction of systemic inflammation following LPS administration, serum concentrations of the proinflammatory cytokines tumor necrosis factor-α (TNF-α) and interleukin-6 (IL-6) were measured. Blood samples were collected from the medial canthus venous plexus 24 h after LPS injection and centrifuged to obtain serum. Serum TNF-α and IL-6 concentrations were quantified using commercially available mouse ELISA kits according to the manufacturers’ instructions. All measurements were performed in duplicate, and cytokine concentrations were calculated from standard curves generated using recombinant cytokine standards.

### Intestinal permeability assessment

Intestinal permeability was assessed using fluorescein isothiocyanate (FITC)-dextran (4 kDa). Mice were fasted for 4 h before FITC-dextran administration while maintaining free access to water. At 20 h after LPS injection, FITC-dextran (600 mg/kg body weight) was administered by oral gavage. Four hours later, corresponding to 24 h after LPS challenge, blood samples were collected and mice were sacrificed for intestinal tissue collection. Serum was separated by centrifugation, and fluorescence intensity was measured using a fluorescence microplate reader at excitation/emission wavelengths of 480/520 nm. FITC-dextran concentrations were determined using a standard curve generated from serial dilutions of FITC-dextran. Increased serum FITC-dextran levels were interpreted as increased intestinal permeability.

### Tissue collection

To ensure consistency among samples, intestinal tissues were collected from the middle-to-proximal segment of the small intestine. Tissue samples were divided into two portions: one portion was fixed in 4% paraformaldehyde for histological, immunohistochemical, and immunofluorescence analyses, whereas the remaining portion was snap-frozen in liquid nitrogen and stored at −80 °C for RNA and protein analyses.

### Experimental reproducibility

For all *in vitro* experiments, at least three independent biological replicates were performed unless otherwise indicated. Histological evaluation and image quantification were conducted using standardized procedures. The number of biological replicates or animals used in each experiment is indicated in the corresponding figure legends.

### Histological and immunohistochemical analyses

Intestinal tissues were fixed in 10% neutral-buffered formalin, embedded in paraffin, and sectioned. Hematoxylin–eosin (H&E) staining was performed according to standard protocols. For immunohistochemistry (IHC), sections were incubated with primary antibodies against GRHL2, E-cadherin, Claudin-1, Claudin-3, Claudin-4, and Occludin (1:200 dilution), followed by HRP-conjugated secondary antibodies and DAB visualization.

### Immunohistochemical quantification

Immunohistochemical staining was semiquantitatively analyzed using ImageJ software (National Institutes of Health, USA). For each experimental group, intestinal tissues from three mice were analyzed. Three representative non-overlapping microscopic fields were randomly selected from each section and imaged under identical microscope settings at ×200 magnification. The mean staining intensity was quantified using the same analysis parameters for all images within each staining batch. The average value obtained from all analyzed fields was used for statistical analysis.

### RNA sequencing and bioinformatic analysis

Total RNA was extracted from intestinal tissues of control and LPS-treated mice (n = 3 per group) and subjected to transcriptome sequencing on the Illumina platform. Raw sequencing reads were processed using fastp to remove adapter-containing reads, low-quality reads, and reads containing excessive unidentified nucleotides. High-quality clean reads were aligned to the mouse reference genome (mm10) using HISAT2, and transcript assembly and gene expression quantification were performed using StringTie. Across all samples, approximately 36–56 million raw reads were generated per library. The Q30 values exceeded 90% for all samples, with GC contents ranging from approximately 48% to 49%, sequencing error rates below 0.01%, and unique mapping rates of approximately 85%, indicating high-quality sequencing data suitable for downstream analyses.

Sample quality and reproducibility were evaluated using principal component analysis (PCA), Euclidean distance analysis, and hierarchical clustering. Differentially expressed genes (DEGs) between groups were identified using DESeq2 based on raw read counts. *P* values were adjusted using the Benjamini–Hochberg false discovery rate (FDR) method. Genes with |log2 fold change| > 1 and adjusted *P* value (padj) < 0.05 were considered significantly differentially expressed.

Functional enrichment analyses, including Gene Ontology (GO) and Gene Set Enrichment Analysis (GSEA), were performed using clusterProfiler to identify biological processes associated with LPS-induced intestinal injury. Protein–protein interaction (PPI) networks were constructed using the STRING database and visualized using Cytoscape. Genes associated with epithelial junction and adhesion-related biological processes were further examined in combination with differential expression results and published literature to identify candidates for subsequent experimental validation.

### Lentiviral construction and transduction

An shRNA targeting mouse GRHL2 (target sequence: GACACGTACAGCTACAACA) was cloned into the pAAV-U6-shRNA/spgRNAv2.0-CMV-mScarlet-WPRE vector, while a scrambled shRNA sequence (CCTAAGGTTAAGTCGCCCTCG) was used as the negative control. Positive clones were verified by colony PCR and Sanger sequencing. For GRHL2 overexpression, the full-length mouse Grhl2 coding sequence (NM_026496.4) was cloned into the pAAV-CMV-Grhl2-3×FLAG-WPRE vector, and the corresponding empty vector served as the control.

HEK293T cells were co-transfected with lentiviral expression plasmids together with packaging and envelope plasmids. Viral supernatants were collected after 48 h, filtered through 0.45 μm membranes, and concentrated by ultracentrifugation. Caco-2 cells were transduced with lentiviral particles at an MOI of 10 in the presence of polybrene. Transduction efficiency was confirmed by mScarlet fluorescence.

For *in vivo* experiments, mice received tail-vein injections of concentrated lentivirus particles (1 × 10^9^ TU/mL) every 1–2 days. Experimental groups included normal controls, LPS-treated mice, GRHL2-overexpression mice, GRHL2-overexpression controls, GRHL2-knockdown mice, and GRHL2-knockdown controls. Following viral administration, mice were challenged with LPS according to the experimental protocol, and intestinal tissues were collected at the designated time point for histological, permeability, qRT-PCR, immunohistochemical, and protein analyses. Detailed information regarding viral constructs, target sequences, plasmid maps, sequencing validation, control vectors, and viral preparation procedures is provided in Supplementary Material 1.

### RT-qPCR

Total RNA was extracted using TRIzol reagent and reverse-transcribed into cDNA. Quantitative PCR was performed using the CFX96 Real-Time PCR System (Bio-Rad, USA). Relative gene expression was calculated using the 2^−ΔΔCt method with β-actin as the internal control. Primer sequences are shown in **Table 1**.

**Table 1:** Primer sequences used for RT-qPCR in mouse tissues.

Gene	Source	Forward primer (5’→3’)	Reverse primer (5’→3’)	Annealing Tm (°C)
GRHL2	Mouse (tissue)	GATGAGGCCTGGAAGTCATATC	TCTCGAGGAACCTTGTAGTAGT	61 (TmF 66 °C/TmR 64 °C)
CLDN1	Mouse (tissue)	CCTGGGAGTGATAGCAATCTT	TGACAGCCATCCTCATCTTC	57 (TmF 62 °C/TmR 60 °C)
Occludin	Mouse (tissue)	GCTCTTTGGAGGAAGCCTAAA	GCTGCTCTTGGGTCTGTATATC	59 (TmF 62 °C/TmR 66 °C)
CLDN4	Mouse (tissue)	GACCGTCAAGGCCAAGAT	TTGTAGAAGTCGCGGATGAC	53 (TmF 56 °C/TmR 60 °C)
CLDN3	Mouse (tissue)	AACTGCGTACAAGACGAGAC	ACCAGGACACCGGTACTAA	55 (TmF 60 °C/TmR 58 °C)
CDH1 (E-cadherin)	Mouse (tissue)	GGCAGAGTGAGATTTGAAGGA	TCCAGCTTGTGGAGCTTTAG	57 (TmF 62 °C/TmR 60 °C)
β-actin	Mouse (tissue)	GACTTCAACAGCAACTCCCACTC	TAGCCGTATTCATTGTCATACCAG	59 (TmF 60 °C/TmR 58 °C)

Annealing temperature is estimated as min (TmF, TmR) − 3 °C.

### Western blotting

Total protein was extracted using RIPA buffer containing PMSF. Equal amounts of protein were separated by SDS-PAGE and transferred to PVDF membranes. After blocking, membranes were incubated with primary antibodies against GRHL2, E-cadherin, Claudin-1, Claudin-3, Claudin-4, and Occludin, followed by HRP-conjugated secondary antibodies. Protein bands were visualized using enhanced chemiluminescence. Densitometric quantification was performed using ImageJ software, and protein expression levels were normalized to β-actin. Detailed information regarding all antibodies, including supplier information, catalog numbers, dilution ratios, expected molecular weights, and observed molecular weights, is summarized in **Supplementary Table 1**. Uncropped Western blot images from three independent biological replicates are provided in the Supplementary Materials for transparency and validation.

### Statistical analysis

All statistical analyses were performed using GraphPad Prism 9.0 (GraphPad Software, San Diego, CA, USA). Data are presented as mean ± SEM from at least three independent experiments unless otherwise indicated.

Comparisons between two groups were performed using unpaired two-tailed Student’s t-tests. For experiments involving more than two groups, statistical significance was assessed using one-way analysis of variance (ANOVA) followed by Tukey’s multiple-comparisons test. TEER measurements comparing control and LPS-treated Caco-2 cells across multiple time points were analyzed using two-way ANOVA followed by Šídák’s multiple-comparisons test. Time-dependent changes in TEER values within the LPS-treated group were analyzed using one-way ANOVA followed by Dunnett’s multiple-comparisons test versus baseline (0 h).

For RNA-seq analysis, differential gene expression was determined using DESeq2. *P* values were adjusted for multiple testing using the Benjamini–Hochberg false discovery rate (FDR) method, and genes with |log2 fold change| > 1 and adjusted *P* value (padj) < 0.05 were considered significantly differentially expressed. A two-sided *P* < 0.05 was considered statistically significant.

## Results

### LPS induces intestinal barrier dysfunction *in vitro* and *in vivo*

**Figure 1:**
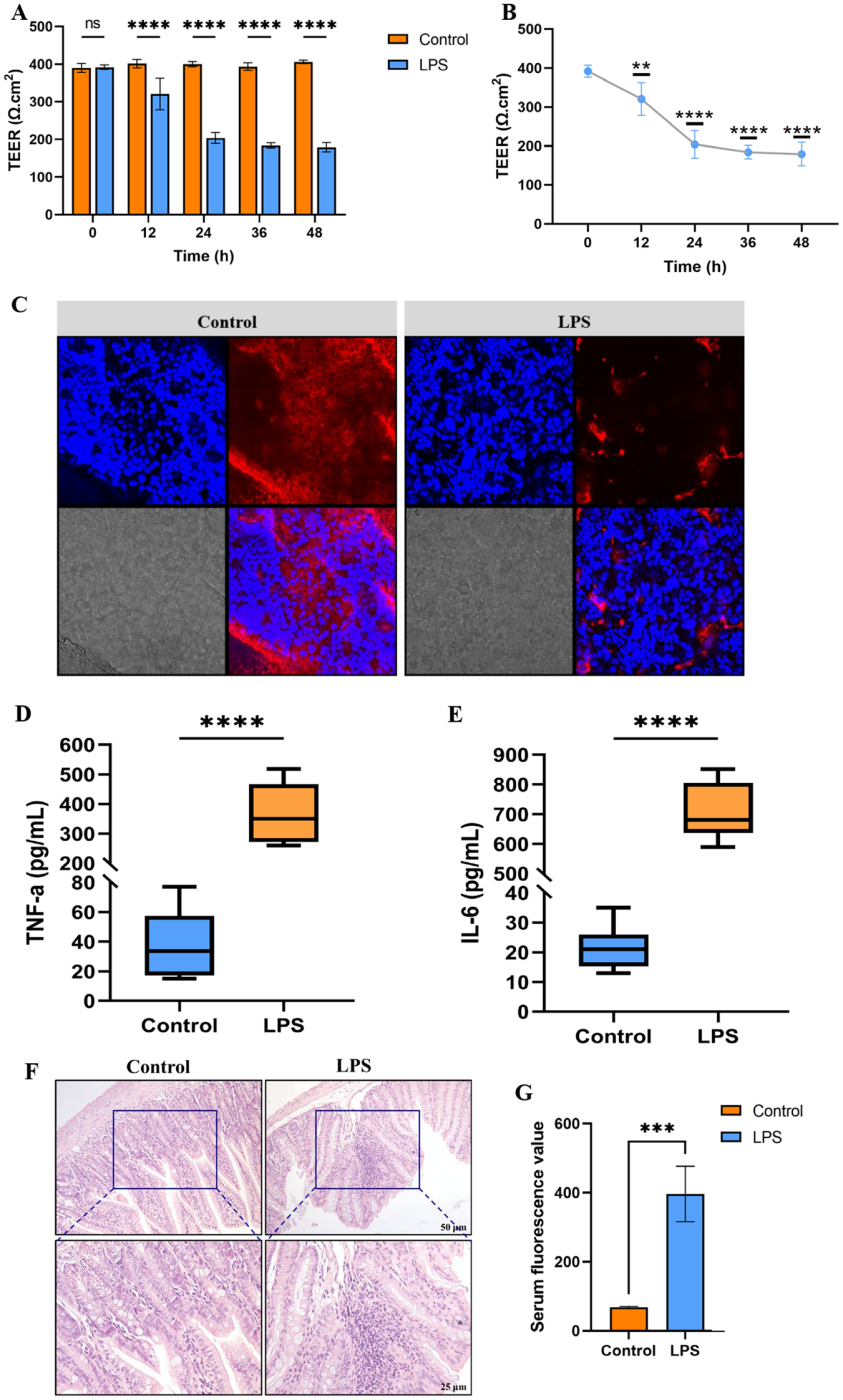
LPS induces disruption of intestinal epithelial tight junctions and barrier dysfunction *in vitro* and *in vivo*. **(A)** Transepithelial electrical resistance (TEER) measurements in Caco-2 cells from the control and LPS-treated groups. **(B)** Dynamic changes in TEER values in LPS-treated Caco-2 cells over 0–48 (h) **(C)** Immunofluorescence staining showing reduced expression and redistribution of Occludin in Caco-2 cells after LPS stimulation. **(D, E)** Serum concentrations of TNF-α **(D)** and IL-6 **(E)** measured by ELISA 24 h after LPS administration. **(F)** Representative H&E staining of intestinal tissues from mice after LPS treatment. **(G)** Comparison of serum fluorescence intensity 2 h after FITC-dextran gavage between the control and LPS-treated mice. Data are presented as mean ± SEM from three independent experiments (n = 3). For **(B)**, statistical significance was analyzed relative to baseline (0 h). **P < 0.01, ***P < 0.001, ****P < 0.0001.

TEER measurements demonstrated that LPS treatment significantly reduced epithelial barrier integrity in Caco-2 monolayers. TEER values progressively decreased at 12, 24, 36, and 48 h after LPS stimulation compared with controls (**Figures 1A, B**). Immunofluorescence staining further revealed disrupted and discontinuous Occludin distribution following LPS treatment, indicating tight junction remodeling (**Figure 1C**). To confirm successful establishment of the LPS-induced inflammatory injury model, serum concentrations of TNF-α and IL-6 were measured 24 h after LPS administration. Both cytokines were significantly elevated in LPS-treated mice compared with control animals, indicating a robust systemic inflammatory response (**Figures 1D, E**).

*In vivo*, H&E staining showed marked epithelial injury and inflammatory cell infiltration in intestinal tissues from LPS-treated mice (**Figure 1F**). FITC-dextran permeability assays demonstrated significantly increased serum fluorescence intensity after LPS treatment, indicating enhanced intestinal permeability (**Figure 1G**).

### Transcriptomic analysis identifies candidate regulators of LPS-induced barrier injury

**Figure 2:**
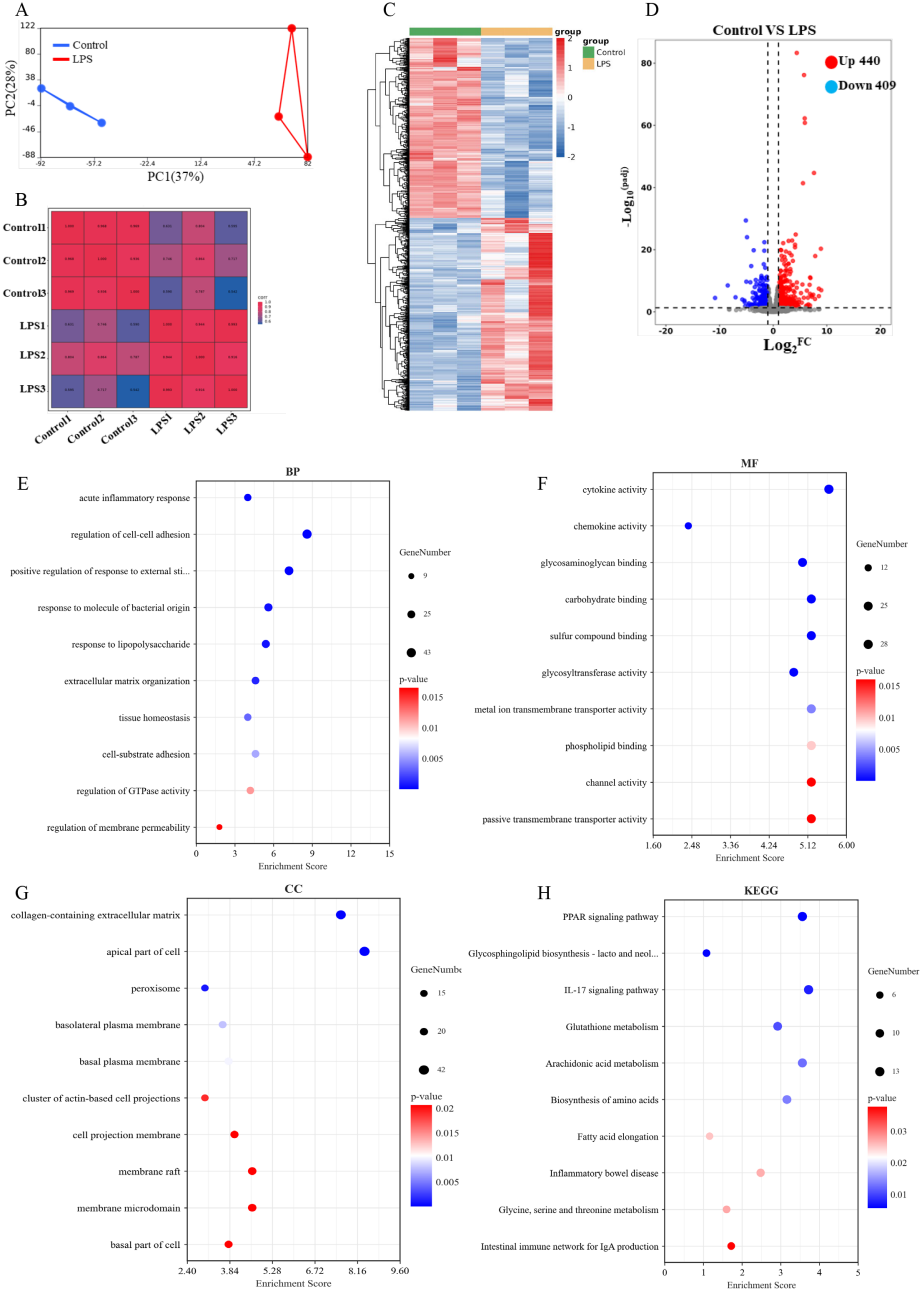
Transcriptomic profiling of intestinal tissues in the LPS-treated and control groups. **(A)** Principal component analysis (PCA) of samples from the LPS-treated and control groups. **(B)** Euclidean distance heatmap of transcriptomic expression profiles among samples. **(C)** Heatmap of differentially expressed genes (DEGs) between the LPS-treated and control groups. **(D)** Volcano plot of DEGs, with upregulated genes shown in red and downregulated genes shown in blue. **(E–G)** Gene Ontology (GO) enrichment analysis of DEGs, including biological process (BP), molecular function (MF), and cellular component (CC). **(H)** KEGG pathway enrichment analysis of DEGs. RNA-seq analysis was performed using three biological replicates per group (n = 3).

RNA sequencing revealed substantial transcriptomic alterations in intestinal tissues following LPS stimulation. Principal component analysis and Euclidean distance clustering demonstrated clear separation between control and LPS-treated samples (**Figures 2A, B**).

A total of 849 differentially expressed genes were identified, including 440 upregulated and 409 downregulated genes (**Figures 2C, D**). GO and KEGG enrichment analyses indicated enrichment of biological processes related to inflammatory responses, cell–cell adhesion, extracellular matrix remodeling, and tissue homeostasis (**Figures 2E–H**).

**Figure 3:**
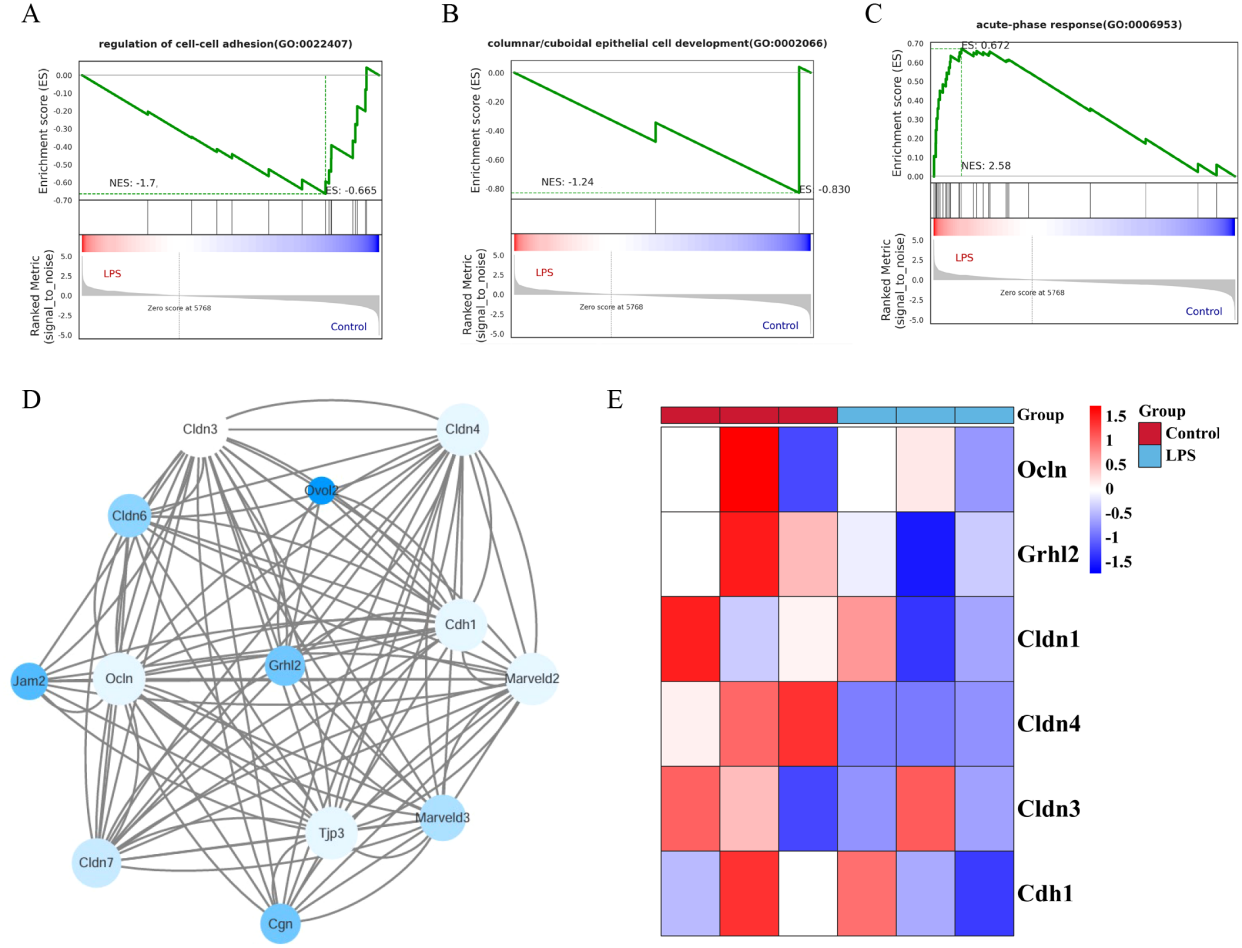
GSEA analysis, PPI network construction, and expression characteristics of junction-associated genes. **(A–C)** GSEA plots comparing the LPS-treated and control groups, showing changes in pathways related to regulation of cell-cell adhesion, epithelial cell development, and the acute-phase response. **(D)** Protein–protein interaction (PPI) network constructed based on differentially expressed genes. **(E)** Heatmap showing the relative expression of junction-associated genes in intestinal tissues from the LPS-treated and control groups. RNA-seq analysis was based on three biological replicates per group (n = 3).

GSEA analysis further demonstrated negative enrichment of pathways related to epithelial development and cell adhesion, whereas acute-phase inflammatory responses were significantly activated (**Figures 3A–C**). PPI network was constructed using differentially expressed genes associated with epithelial junction and adhesion-related processes (**Figure 3D**). GRHL2 was present within the interaction network together with multiple epithelial junction-associated genes. In addition, heatmap analysis showed that several junction-associated genes were downregulated after LPS stimulation (**Figure 3E**).

### GRHL2 expression is reduced in intestinal tissues following LPS exposure

**Figure 4:**
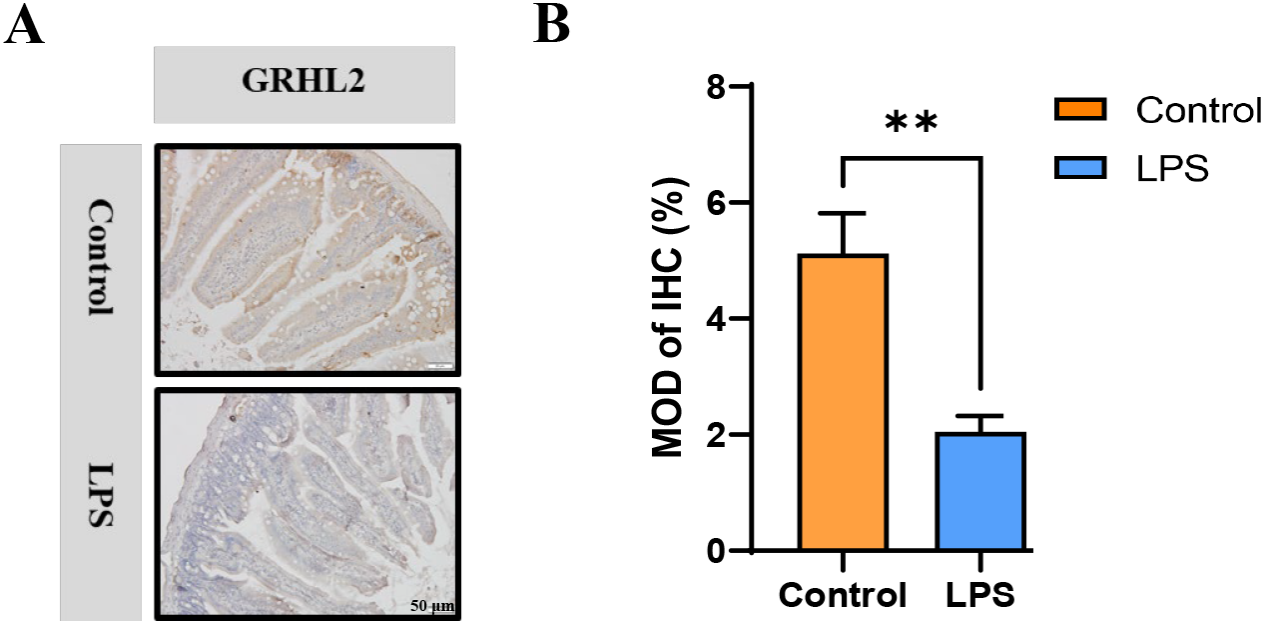
GRHL2 expression is downregulated in mouse intestinal tissues after LPS stimulation. **(A)** Representative immunohistochemical (IHC) staining images of GRHL2 in mouse intestinal tissues. **(B)** Semiquantitative analysis of GRHL2 IHC staining. Original magnification, ×200. Data are presented as mean ± SEM (n = 3 mice per group). **P < 0.01.

**Figure 5:**
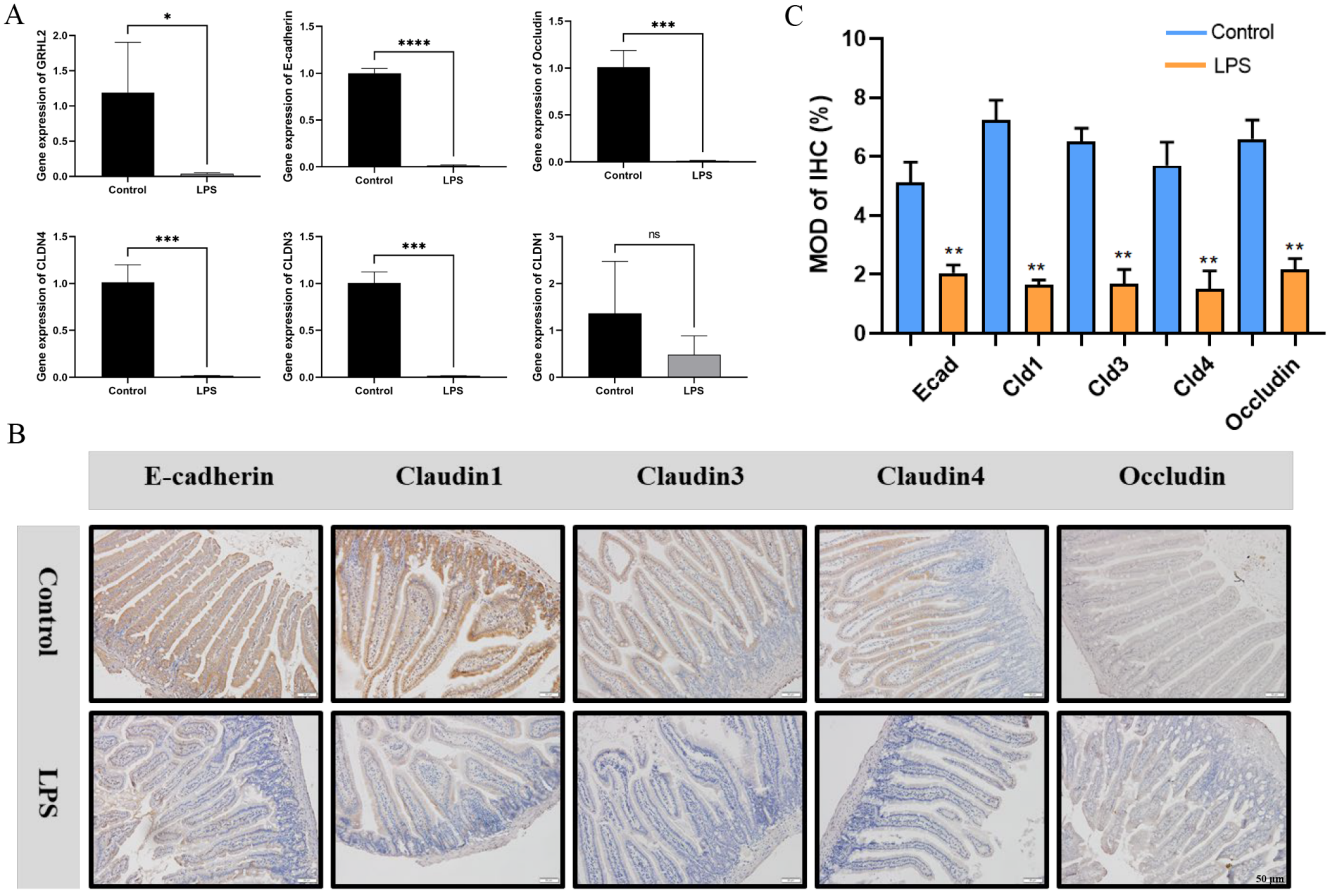
Expression of GRHL2 and epithelial junction-associated molecules in intestinal tissues following LPS exposure. **(A)** qRT-PCR analysis of mRNA expression levels of GRHL2, E-cadherin, Claudin-1, Claudin-3, Claudin-4, and Occludin in intestinal tissues from the LPS-treated and control groups. **(B)** Representative immunohistochemical (IHC) staining images of GRHL2 and junction-associated proteins in mouse intestinal tissues. **(C)** Semiquantitative analysis of IHC staining. Original magnification, ×200. Data are presented as mean ± SEM (n = 3 mice per group). *P < 0.05, **P < 0.01, ***P < 0.001, ****P < 0.0001.

Immunohistochemical analysis showed that GRHL2 expression was significantly decreased in intestinal tissues after LPS stimulation (**Figures 4A, B**). qRT-PCR further confirmed that the mRNA expression levels of GRHL2, E-cadherin, Claudin-3, Claudin-4, and Occludin were markedly reduced in LPS-treated mice (**Figure 5A**). IHC staining also demonstrated reduced expression of GRHL2 and multiple junction- associated proteins in the LPS-treated group (**Figures 5B, C**).

**Figure 6:**
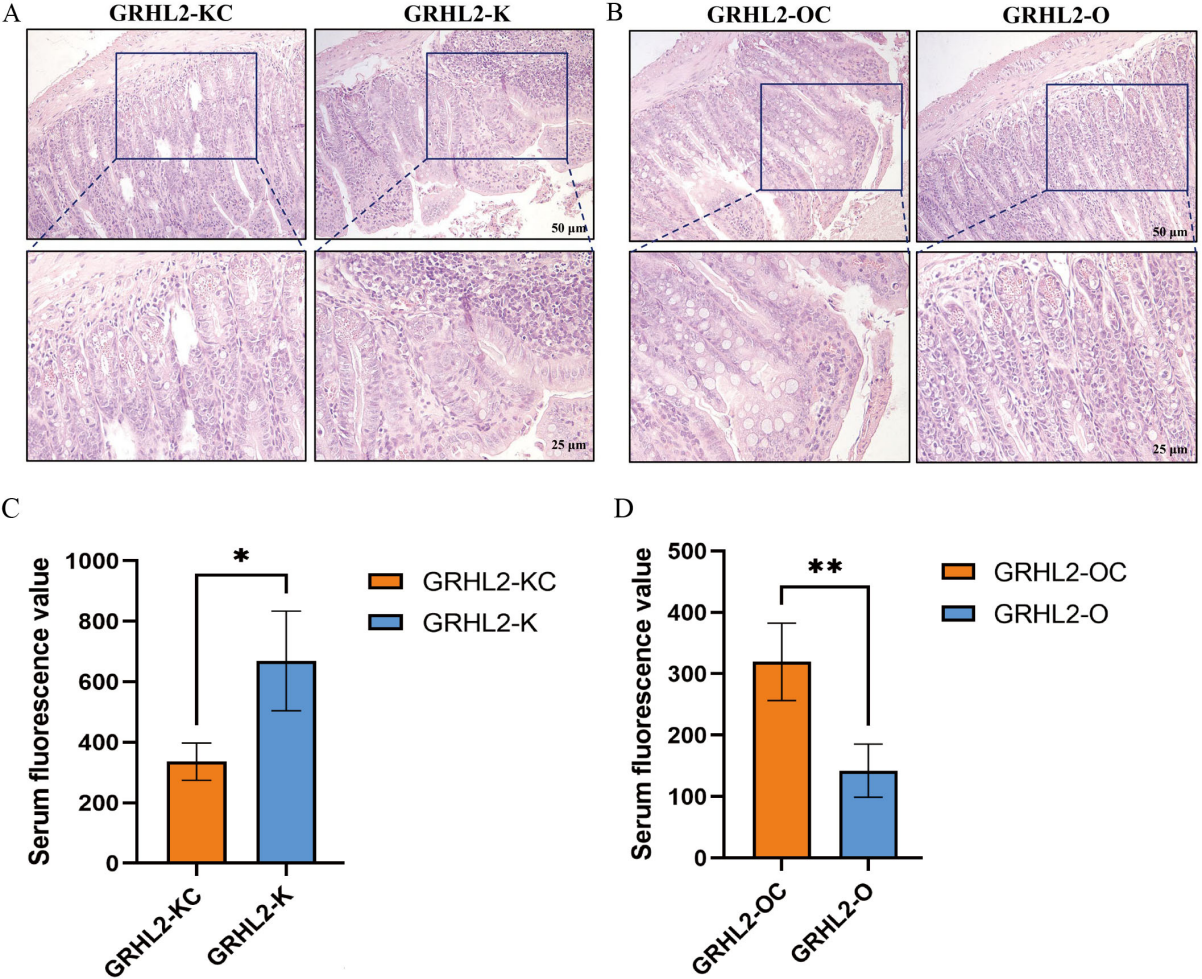
Effects of GRHL2 knockdown or overexpression on LPS-induced intestinal injury and permeability in mice. **(A)** Representative H&E staining of small intestinal tissues from mice with GRHL2 knockdown under LPS stimulation. **(B)** Representative H&E staining of small intestinal tissues from mice with GRHL2 overexpression under LPS stimulation. **(C)** Serum fluorescence intensity measured 2 h after FITC-dextran gavage in mice with GRHL2 knockdown following LPS stimulation. **(D)** Serum fluorescence intensity measured 2 h after FITC-dextran gavage in mice with GRHL2 overexpression following LPS stimulation. Data are presented as mean ± SEM (n = 3 mice per group). Statistical significance was analyzed using one-way ANOVA followed by Tukey’s multiple-comparisons test. *P < 0.05, **P < 0.01.

To further investigate the functional role of GRHL2, *in vivo* lentiviral systems were used to modulate GRHL2 expression. GRHL2 knockdown aggravated intestinal epithelial injury and inflammatory infiltration after LPS treatment, whereas GRHL2 overexpression alleviated histological damage (**Figures 6A, B**).

**Figure 7:**
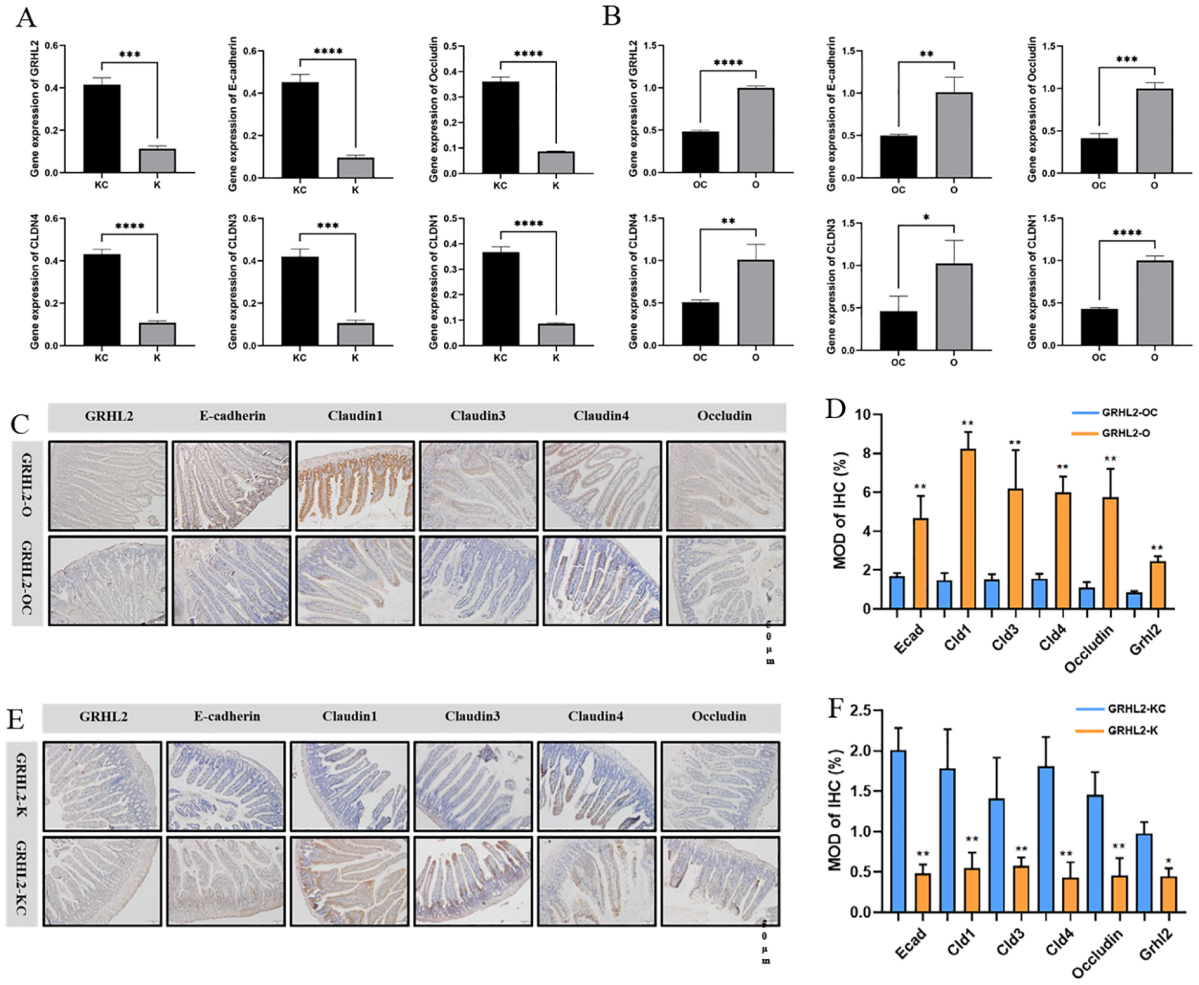
Association between GRHL2 modulation and expression of epithelial junction-associated molecules in mouse intestinal tissues. **(A)** qRT-PCR analysis of mRNA expression levels of GRHL2, E-cadherin, Claudin-1, Claudin-3, Claudin-4, and Occludin in mouse intestinal tissues after GRHL2 knockdown. **(B)** qRT-PCR analysis of mRNA expression levels of the above genes in mouse intestinal tissues after GRHL2 overexpression. **(C, D)** Representative immunohistochemical (IHC) staining images of GRHL2 and junction-associated proteins in mouse intestinal tissues after GRHL2 knockdown. **(E, F)** Representative immunohistochemical (IHC) staining images of GRHL2 and junction-associated proteins in mouse intestinal tissues after GRHL2 overexpression. Original magnification, ×200. Data are presented as mean ± SEM (n = 3 mice per group). Statistical significance was analyzed using one-way ANOVA followed by Tukey’s multiple-comparisons test. *P < 0.05, **P < 0.01, ***P < 0.001, ****P < 0.0001 versus the control group.

**Figure 8:**
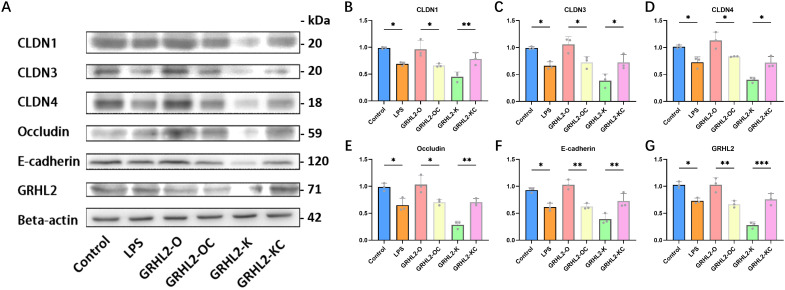
Western blot analysis and quantitative assessment of GRHL2 and epithelial junction-associated proteins. **(A)** Representative Western blot images of CLDN1, CLDN3, CLDN4, Occludin, E-cadherin, and GRHL2 in mouse intestinal tissues under different experimental conditions. **(B–G)** Quantitative densitometric analysis of CLDN1 **(B)**, CLDN3 **(C)**, CLDN4 **(D)**, Occludin **(E)**, E-cadherin **(F)**, and GRHL2 **(G)** protein expression normalized to β-actin. Data are presented as mean ± SEM (n = 3 mice per group). Statistical significance was analyzed using one-way ANOVA followed by Tukey’s multiple-comparisons test. *P < 0.05, **P < 0.01, ***P < 0.001.

Consistently, FITC-dextran assays showed increased intestinal permeability in GRHL2-knockdown mice and reduced permeability in GRHL2-overexpression mice (**Figures 6C, D**). qRT-PCR and IHC analyses showed that alterations in GRHL2 expression were accompanied by corresponding changes in the expression of multiple junction-related molecules, including E-cadherin, Claudin-1, Claudin-3, Claudin-4, and Occludin (**Figures 7A–F**). Western blot analysis further confirmed these findings at the protein level (**Figure 8**).

### Association between GRHL2 downregulation and intestinal barrier dysfunction

**Figure 9:**
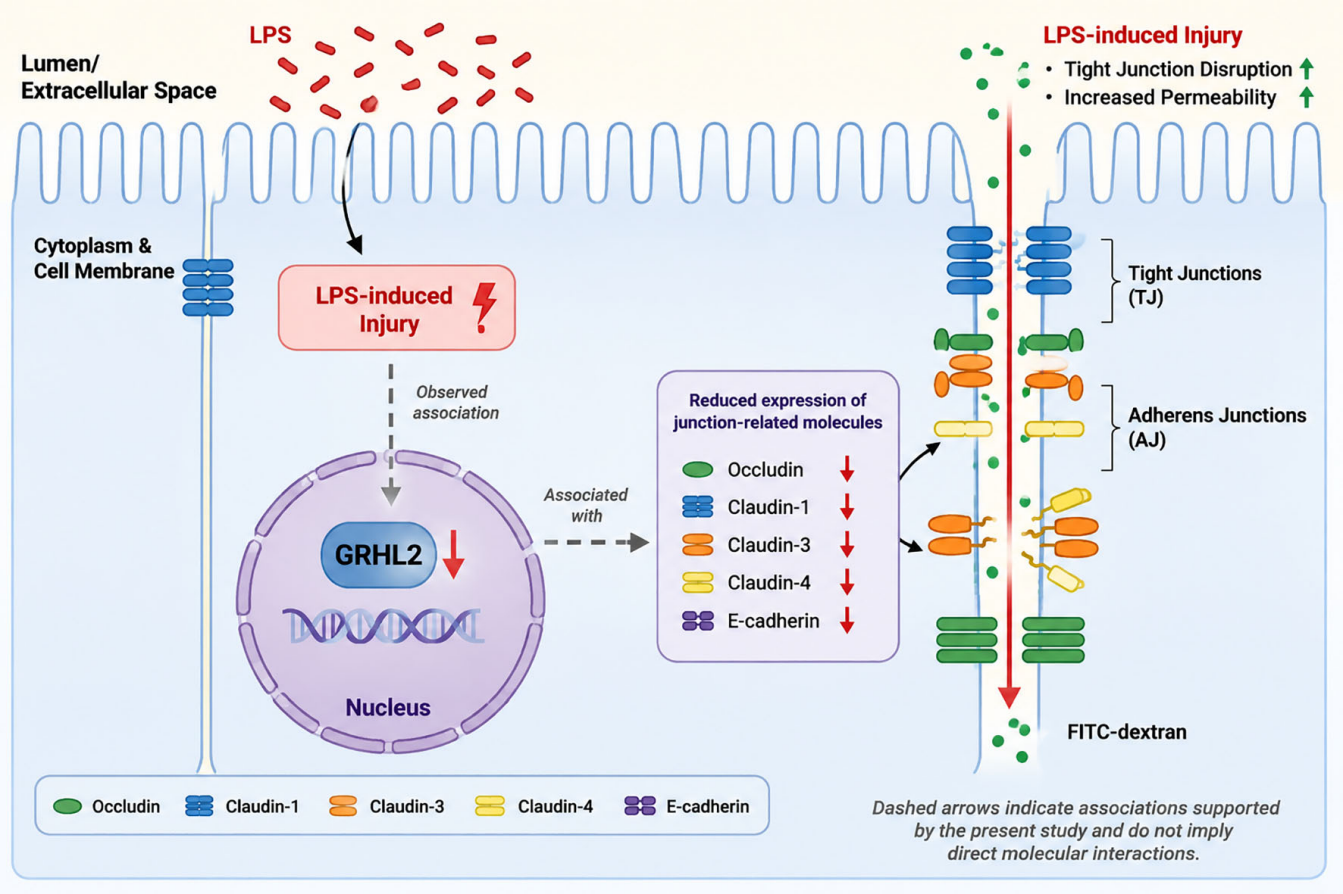
Summary of the observed association between GRHL2 downregulation and intestinal barrier dysfunction following LPS stimulation. In the present study, LPS challenge was associated with decreased GRHL2 expression in intestinal tissues, accompanied by reduced expression of Occludin, Claudin-1, Claudin-3, Claudin-4, and E-cadherin. These changes were observed together with disruption of epithelial junctional structures and increased intestinal permeability, as evidenced by enhanced FITC-dextran translocation. The schematic summarizes the experimental findings of this study and does not propose a specific upstream signaling pathway or direct molecular mechanism linking LPS stimulation to GRHL2 downregulation.

Based on transcriptomic and functional analyses, a summary model was proposed (**Figure 9**). LPS stimulation was associated with reduced GRHL2 expression and decreased expression of several junction-related molecules, accompanied by impaired epithelial barrier integrity. These findings suggest that GRHL2 contributes to the maintenance of intestinal epithelial homeostasis and may represent a potential protective factor against LPS-induced barrier dysfunction.

## Discussion

In the present study, we investigated the involvement of GRHL2 in LPS-induced intestinal epithelial barrier dysfunction. Sepsis remains a life-threatening syndrome with substantial mortality and is characterized by a dysregulated host response to infection that can lead to multiple organ dysfunction, including gastrointestinal injury (10). Consistent with previous reports, intraperitoneal administration of LPS induced a systemic inflammatory response, as reflected by increased serum TNF-α and IL-6 levels, and resulted in marked intestinal epithelial injury in our experimental model. *In vitro*, LPS exposure impaired epithelial barrier integrity, as demonstrated by reduced TEER values and disrupted Occludin distribution in Caco-2 monolayers. *In vivo*, LPS administration caused epithelial injury, inflammatory cell infiltration, and increased FITC-dextran leakage into the circulation. These findings are consistent with the concept that sepsis- associated intestinal injury compromises intestinal epithelial integrity and increases paracellular permeability (7). Together, the *in vitro* and *in vivo* data confirmed successful establishment of LPS-induced intestinal barrier dysfunction models.

Transcriptomic analysis provided further insight into the biological processes associated with LPS-induced intestinal injury. Consistent with the histological and permeability changes observed in our experimental models, RNA-seq analysis demonstrated clear transcriptional differences between control and LPS-treated intestinal tissues. Functional enrichment analyses revealed significant changes in biological processes related to inflammatory responses, regulation of cell–cell adhesion, extracellular matrix organization, and tissue homeostasis. Furthermore, GSEA demonstrated suppression of epithelial development and cell–cell adhesion programs together with enrichment of acute-phase inflammatory responses. These findings suggest that LPS-induced intestinal injury is accompanied by coordinated alterations in both inflammatory and epithelial biological processes, supporting the concept that disruption of epithelial homeostasis represents an important component of intestinal barrier dysfunction during inflammatory injury.

Among the genes altered following LPS exposure, GRHL2 attracted particular attention because of its established role in epithelial biology (11). GRHL2 is a member of the Grainyhead-like family of transcription factors and has been implicated in epithelial differentiation, maintenance of epithelial identity, and preservation of tissue homeostasis in multiple organs (12). Previous studies have also suggested that GRHL2 is associated with epithelial barrier function and cellular junction organization under both physiological and pathological conditions (5–9). We found that GRHL2 expression was significantly reduced in intestinal tissues after LPS exposure, indicating that GRHL2 may participate in the epithelial response to inflammatory injury. This observation is in line with previous evidence showing that GRHL2 may be targeted during epithelial barrier disruption in other mucosal contexts, such as Porphyromonas gingivalis-induced oral epithelial barrier injury (7). Therefore, based on both the transcriptomic findings and the known biological functions of GRHL2, we selected GRHL2 for further investigation in the context of LPS-induced intestinal barrier dysfunction.

Functional modulation of GRHL2 further supported its protective role in the intestinal barrier. GRHL2 knockdown aggravated LPS-induced histological injury and increased intestinal permeability, whereas GRHL2 overexpression attenuated tissue damage and reduced FITC-dextran leakage. These findings indicate that alterations in GRHL2 expression are closely associated with the severity of barrier dysfunction in the LPS model. Importantly, the reciprocal phenotypes observed following GRHL2 knockdown and overexpression provide functional evidence supporting a beneficial role for GRHL2 in preserving intestinal epithelial integrity under inflammatory conditions. Together, these results support a functional association between GRHL2 expression and maintenance of intestinal barrier homeostasis during LPS-induced injury.

The association between GRHL2 expression and alterations in junction-related molecules observed in the present study is biologically meaningful given the central role of epithelial junctions in barrier maintenance. Tight junctions and adherens junctions form an integrated structural and signaling network that controls paracellular permeability and epithelial polarity (6). Occludin and claudins contribute directly to tight junction sealing, whereas E-cadherin-mediated adherens junctions provide essential support for epithelial architecture and junctional stability. Previous studies have shown that claudin family members are essential for epithelial barrier homeostasis in multiple organs, including the skin and stomach (13–16). In the present study, LPS exposure was accompanied by reduced expression of several junction-related molecules at the mRNA and protein levels, and these changes were partially reversed by GRHL2 overexpression while becoming more pronounced following GRHL2 knockdown. These findings suggest that GRHL2 expression is closely associated with the maintenance of epithelial junctional integrity during inflammatory injury. However, because direct promoter binding, chromatin occupancy, and transcriptional regulatory activity were not examined, the present study does not establish whether these molecules represent direct downstream targets of GRHL2. Instead, our findings support an association between GRHL2 expression and the preservation of epithelial junctional homeostasis under conditions of LPS-induced injury.

The mechanisms responsible for GRHL2 downregulation during LPS-induced intestinal injury remain unclear. Previous studies have shown that LPS-induced barrier dysfunction involves multiple interconnected pathways, including inflammatory signaling cascades, oxidative stress responses, cytoskeletal remodeling, and alterations in epithelial junction organization. TLR4/NF-κB- and MAPK-related pathways have been widely implicated in intestinal inflammation and barrier disruption, while MLCK-mediated cytoskeletal contraction has been reported to contribute to increased epithelial permeability (17, 18). However, these signaling pathways were not directly examined in the present study, and therefore no conclusions can be drawn regarding their involvement in GRHL2 regulation. Based on our findings, we propose only that GRHL2 downregulation is associated with intestinal barrier dysfunction during LPS-induced injury. Future studies incorporating pathway inhibition, genetic manipulation, and chromatin-level analyses will be required to determine the upstream mechanisms responsible for GRHL2 suppression and to clarify how GRHL2 participates in epithelial barrier regulation under inflammatory conditions.

Several limitations should be acknowledged. First, although GRHL2 knockdown and overexpression experiments support a functional role for GRHL2 in intestinal barrier regulation, the present study does not establish whether GRHL2 directly binds to the promoters or enhancers of junction-associated genes. Future studies using chromatin immunoprecipitation, promoter reporter assays, or single-cell transcriptomic approaches are needed to clarify the direct transcriptional targets of GRHL2 in intestinal epithelial cells. Second, the upstream mechanisms by which LPS suppresses GRHL2 remain unresolved. Although inflammatory pathways such as TLR4/NF-κB and MAPK signaling have been implicated in intestinal barrier injury, their relationship with GRHL2 was not examined in the current study. Third, RNA and protein analyses were performed using whole intestinal tissues rather than isolated intestinal epithelial cells. Therefore, we cannot completely exclude the possibility that some of the observed expression changes were influenced by epithelial loss or villus injury following LPS exposure. Fourth, *in vivo* GRHL2 modulation was achieved through systemic viral delivery, and epithelial-specific transduction efficiency was not directly validated. Consequently, the observed effects should be interpreted as the result of whole-intestinal GRHL2 modulation rather than definitive epithelial-specific regulation. Finally, the present study employed an LPS-induced endotoxemia model, which reproduces important aspects of inflammatory injury but does not fully recapitulate the complexity of clinical sepsis. Future studies using epithelial-specific models, intestinal organoids, clinically relevant sepsis models, and human samples will help further clarify the role of GRHL2 in intestinal barrier dysfunction.

In conclusion, our study demonstrates that LPS induces intestinal epithelial barrier dysfunction accompanied by GRHL2 downregulation and decreased expression of several junction-related molecules. Functional manipulation of GRHL2 indicates that GRHL2 contributes to the preservation of intestinal barrier integrity during LPS-induced injury. These findings improve our understanding of the association between GRHL2 expression and intestinal barrier dysfunction during inflammatory injury.

## Data Availability

The data analyzed in this study is subject to the following licenses/restrictions: The original raw dataset is available upon reasonable request from the corresponding author. Requests to access these datasets should be directed to zhangdong@jlu.edu.cn.
