# Supplementary material for: GRHL2 contributes to the maintenance of intestinal epithelial barrier integrity during LPS-induced injury
Source: Front Immunol. 2026 Jul 15;17:1881734. doi: 10.3389/fimmu.2026.1881734 (PMC13414807; doi:10.3389/fimmu.2026.1881734)
Supplement: Supplementary file 1 [file Table1.docx]

**Supplementary Material**

**Plasmid Construction Report: HY-AAV202303-606 (Y25736)**

**Abstract:** Short hairpin RNA (shRNA) targeting mouse Grhl2 (Gene ID: NM_026496.4) was designed and cloned into an adeno-associated virus (AAV) vector. This vector facilitates RNA interference (RNAi)-mediated knockdown of GRHL2 gene expression and can be utilized for both transient transfection in cell cultures and AAV-mediated gene silencing in animal models.

**Keywords:** GRHL2, RNAi, shRNA

**Experimental Principle:** RNA interference (RNAi) is a conserved biological process where double-stranded RNA (dsRNA) induces specific degradation of homologous mRNA. Short hairpin RNA (shRNA), driven by RNA polymerase III promoters (such as U6 and H1), is processed intracellularly into RNA-induced silencing complex (RISC) to mediate target mRNA cleavage.

**Experimental Steps:**

1. **shRNA Design and Oligonucleotide Synthesis:** shRNA sequences were designed, and oligonucleotides were synthesized.
2. **Annealing of Oligonucleotides:** Single-stranded oligonucleotides were annealed to form double-stranded oligonucleotides with sticky ends by heating at 95°C for 5 minutes and gradually cooling to room temperature.
3. **Vector Linearization:** The pAAV-U6-shRNA/spgRNAv2.0-CMV-mScarlet-WPRE vector was linearized by restriction enzyme digestion (NheI and BamHI).
4. **Ligation Reaction:** Annealed oligonucleotides were ligated into the linearized vector, replacing the original ccdB toxicity gene.
5. **Transformation:** DH5α competent cells were transformed with the ligation products.
6. **Colony PCR Screening:** Transformed colonies were screened using colony PCR.
7. **Sequencing Validation:** Positive colonies were verified by Sanger sequencing using CMV-F (5’-CGCAAATGGGCGGTAGGCGTG-3’) and WPRE-R (5’-CATAGCGTAAAAGGAGCAACA-3’) primers.
8. **Plasmid Extraction:** Correctly sequenced clones underwent high-purity plasmid extraction.

**Constructed shRNA Sequences:** - Y25736 Target Sequence: GACACGTACAGCTACAACA - Y14305 (Control) Target Sequence: CCTAAGGTTAAGTCGCCCTCG

**Lentiviral Vector Production and Transduction:** HEK293T cells were co-transfected with lentiviral vectors along with packaging and envelope plasmids. Viral supernatants were harvested after 48 hours, filtered through 0.45 µm membranes, and concentrated by ultracentrifugation. Caco-2 cells were transduced with lentivirus in the presence of polybrene, and media was refreshed after 24-48 hours. Transduction was verified by transgene or marker expression.

**In Vivo Application:** Mice were administered lentivirus (1 × 10^9 infectious units) via tail vein injections at intervals of 1-2 days. The experimental groups included: 1. Normal mice 2. Sepsis model 3. GRHL2-overexpression sepsis model 4. GRHL2-overexpression control 5. GRHL2-knockdown sepsis model 6. GRHL2-knockdown control

**Plasmid Details (H29817):** - Clone Number: H29817 - Gene Name: Grhl2 (GenBank ID: NM_026496.4) - Species: Mouse - Restriction Sites: NheI, BamHI - Prokaryotic Resistance: Ampicillin - Vector: AOV021 pAAV-CMV-MCS-3xFLAG-WPRE - Final Construct: pAAV-CMV-Grhl2-3xFLAG-WPRE

**Plasmid Map Components:** - 3xFLAG tag - Left and Right Inverted Terminal Repeats (L-ITR, R-ITR) - Human Growth Hormone (hGH) polyadenylation signal - Ampicillin Resistance (AmpR) - Woodchuck Hepatitis Virus Posttranscriptional Regulatory Element (WPRE) - Cytomegalovirus (CMV) Promoter - Origins: f1 origin, pUC origin

**Sequencing Results:** - Y25736 shRNA insert sequence verification successful (completely consistent with the design sequence). - Y14305 control sequence verification successful (completely consistent with the design sequence).

**References:** F.M. Ausubel et al., Current Protocols in Molecular Biology (4th Edition), Science Press.

**In Vivo Viral Administration and Experimental Design:** For in vivo studies, mice received tail-vein injections of concentrated lentiviral particles (1 × 10⁹ TU/mL) carrying either GRHL2-targeting shRNA, scrambled shRNA control vectors, GRHL2-overexpression vectors, or corresponding control vectors. Viral administration was performed at 1–2 day intervals according to the experimental protocol.

The following experimental groups were included:

1. Normal control group
2. LPS-induced injury group
3. GRHL2-overexpression + LPS group
4. Overexpression vector control + LPS group
5. GRHL2-knockdown + LPS group
6. Scrambled shRNA control + LPS group

Following viral administration, mice were challenged with lipopolysaccharide (LPS) to induce intestinal injury. Intestinal tissues were subsequently harvested for histological analysis, permeability assessment, immunohistochemistry, qRT-PCR, and Western blotting.

The present study was designed to evaluate the association between altered GRHL2 expression and intestinal barrier dysfunction. Cell-type-specific transduction efficiency within intestinal epithelial cells was not directly assessed.

**Supplementary Table1. Antibody Information and Observed Molecular Weights for Western Blot Analysis**

| Protein | Supplier | Catalog No. | Dilution | Expected MW (kDa) | Observed Bands (kDa) |
| --- | --- | --- | --- | --- | --- |
| Claudin-1 (CLDN1) | Invitrogen | 34-4900 | 1:1000 | ~20 | ~20 |
| Claudin-3 (CLDN3) | Invitrogen | 34-1700 | 1:1000 | ~20 | ~18–20 |
| Claudin-4 (CLDN4) | Invitrogen | 32-9400 | 1:1000 | ~18 | ~18–23 |
| GRHL2 | Abcam | ab271023 | 1:1000 | ~71 | ~71 |
| Occludin | Abcam | ab216327 | 1:1000 | ~59 | ~59 (full-length); additional bands at ~65, ~53, ~25, ~23 |
| E-cadherin (CDH1) | Abcam | ab231303 | 1:1000 | ~97 (datasheet) | ~120–130 |

**Notes:** Occludin quantification was performed using the full-length band at approximately 59 kDa. E-cadherin migrated at approximately 120–130 kDa, consistent with the known glycosylated form of the protein. Uncropped membranes from three independent biological replicates are provided as supplementary materials.
